# Molecular Crosstalk in Age-Related Macular Degeneration: Integrating Oxidative Stress, Inflammation, microRNAs, and Genetic Susceptibility Toward Precision Therapeutics

**DOI:** 10.3390/biom16020234

**Published:** 2026-02-03

**Authors:** Charlotte Delrue, Reinhart Speeckaert, Marijn M. Speeckaert

**Affiliations:** 1 Department of Nephrology, Ghent University Hospital, 9000 Ghent, Belgium; charlotte.delrue@ugent.be; 2 Department of Dermatology, Ghent University Hospital, 9000 Ghent, Belgium; reinhart.speeckaert@uzgent.be; 3 Research Foundation-Flanders (FWO), 1000 Brussels, Belgium

**Keywords:** age-related macular degeneration, complement dysregulation, mitochondrial dysfunction, oxidative stress, microRNA

## Abstract

Age-related macular degeneration (AMD) is an increasingly prevalent source of permanent visual impairment in the aging population and is widely accepted as a multi-factorial neurodegenerative disorder of the retina. While there has been significant progress in treating neovascular AMD, there are currently no effective disease-sparing treatments for dry AMD and geographic atrophy. To date, research has begun to reveal the complex relationship between the environment and genetic predisposition in AMD pathogenesis. Various environmental factors responsible for AMD include oxidative stress, mitochondrial dysfunction, inflammation, abnormal complement activation, and epigenetic regulation, which interact dynamically to drive disease progression. This review summarizes recent data and provides a comprehensive model for understanding how these interacting factors lead to the progression of AMD from an early stage to advanced stages with complications associated with the disease. We highlight the central role of retinal pigment epithelial mitochondrial failure and impaired stress resilience as upstream drivers that amplify inflammation and complement-mediated injuries. We also discuss how dysregulated miRNAs and proteomic network remodeling contribute to disease heterogeneity. Emerging therapeutic strategies are reviewed in the context of molecular endotyping and personalized intervention. Finally, we outline future directions toward precision medicine in AMD, emphasizing early disease modification, rational combination therapies, and the need to bridge the translational gaps between molecular discovery and clinical trial design.

## 1. Introduction

Age-related macular degeneration (AMD) is a progressive degenerative disorder of the retina. It particularly affects the central retina. It is one of the principal global causes of irreversible vision loss in individuals aged >55 years. The number of people with AMD continues to grow globally due to an aging population. By 2040, projections indicate that there will be approximately 300 million people with ocular AMD, creating an increasing socioeconomic burden on healthcare systems, particularly in developed countries [1,2,3]. Clinically, AMD causes increasing central vision loss, which results from the degeneration of the retinal pigment epithelium (RPE), photoreceptors, Bruch’s membrane, and the capillary network beneath it, ultimately resulting in an impaired macula.

AMD has traditionally been divided into three stages: early and intermediate (those with observable physiological changes) and late, which can be further classified as either neovascular AMD (nAMD) or geographic atrophy (GA). Much progress has been made in the treatment of nAMD via vascular endothelial growth factor (VEGF)-inhibiting therapies. However, very little evidence exists regarding the use of disease-modifying medications for the treatment of GA or dry AMD that will provide patients with any substantial benefit [4,5,6]. In addition, during the middle stage of AMD development, people with dry AMD or GA may remain asymptomatic for long periods. Few patients exhibit any major external visible signs of damage until they transition into the late intermediate stage. Understanding the early pathophysiological changes leading to the beginning of AMD progression and accurately determining viable therapeutic targets once the patient reaches the initial intermediate stage will ultimately lead to the availability of new and/or improved treatment alternatives for patients during the initiation of AMD development.

From a biological viewpoint, AMD serves as an archetype of a multifactorial and complex disease that results from interactions among multiple genetic risk factors, epigenetic regulation, environmental exposures, and age-related degenerative changes to cellular function. To date, genome-wide association studies (GWAS) have identified over 50 loci on the human genome associated with AMD, most of which fall under several broader categories of genes and include pathways involved in the activation of the complement system and lipid metabolism, as well as remodeling of the extracellular matrix (ECM) and regulation of inflammation [7]. Polymorphisms found in the complement gene family (e.g., *CFH*, *C3*, *CFI*, and *CFB*) and the alleles at the age-related maculopathy susceptibility 2 (ARMS2)/high-temperature requirement A serine peptidase 1 (HTRA1) locus are among the strongest genetic risk factors for developing AMD, illustrating that chronic dysregulation of the innate immune system is a central mechanism contributing to AMD development.

The initiation of retinal damage and its associated longevity, oxidative stress, is a leading contributor to retinal deterioration beyond genetic predisposition. Due to the high metabolic demands of the macula, continuous light exposure, increased oxygen use, and abundance of unsaturated fatty acids, it is extremely sensitive to oxidative stress. Age-related mitochondrial dysfunction in RPE cells results in energetic dysmetabolism of energy source molecules, increased production of reactive oxygen species (ROS), thereby predisposing the retina to progressive degeneration [2,8,9,10]. However, oxidative stress exacerbates inflammatory signaling and activates the complement cascade to form a self-perpetuating cycle of retinal degeneration.

Chronic low-grade inflammation, also known as para-inflammation, is a primary characteristic of AMD. Initially, para-inflammation acts as a defense mechanism. However, due to a prolonged period of continued activation of the inflammatory state, it changes from a protective to a harmful role, often contributing to the formation of drusen, dysfunction of the RPE, and loss of photoreceptors [11,12]. Drusen are now considered biologically active substances that contain high concentrations of complement, lipids, amyloid-associated proteins, and inflammatory mediators. Drusen act as both a biomarker and an active participant in the progression of the disease [13]. The shift from para-inflammation being regulated marks the advancement of a patient’s AMD status from intermediate to advanced.

Recent studies focus primarily on the regulatory function of microRNAs (miRNAs), which have been identified as methylation-based regulators of AMD, via post-transcriptional modulation of gene expression and also as mediators of control over oxidative stress responses, inflammatory cascades, angiogenesis, and maintenance of mitochondrial homeostasis [14,15]. miRNA profiles that are dysregulated are present in retinal tissue, RPE cells (human donor and in vitro studies), and circulating fluids of AMD patients (observational clinical studies). These findings may represent both a pathogenic mechanism associated with AMD and a potential source of a non-invasive biomarker. These findings will allow for expanded avenues of research into the development of new therapeutic strategies targeting the restoration of molecular homeostasis, and not on the targeting of single downstream effectors.

An explosion in technology advancements within high-throughput genomic, transcriptomic, proteomic, and metabolomic platforms has further pushed our understanding of AMD from a simple linear pathophysiological pathway towards AMD as a network disease. The systems biologic paradigm has uncovered many layers of interference between oxidative stress pathways, complement activation, dyslipidemia, and mitochondrial damage, thus creating the need for integrative therapeutic approaches in AMD [16]. This changing paradigm has also influenced contempory therapeutic development strategies. Several therapies that are currently evaluated include those targeting upstream pathways [i.e., complement inhibitors, gene-based gene transfer methods, and gene editing tools such as the clustered regularly interspaced short palindromic repeats (CRISPR)/CRISPR-associated (Cas) system] rather than targeting complications that have manifested themselves when the disease is already in an advanced state.

In this context, elucidating the molecular determinants that govern disease initiation, progression, and phenotypic divergence between nAMD and GA is essential for the development of precision medicine approaches. Identifying molecular signatures predictive of progression from intermediate AMD to advanced stages could enable earlier intervention, personalized risk stratification, and combination therapies tailored to individual pathogenic profiles.

**Figure 1:**
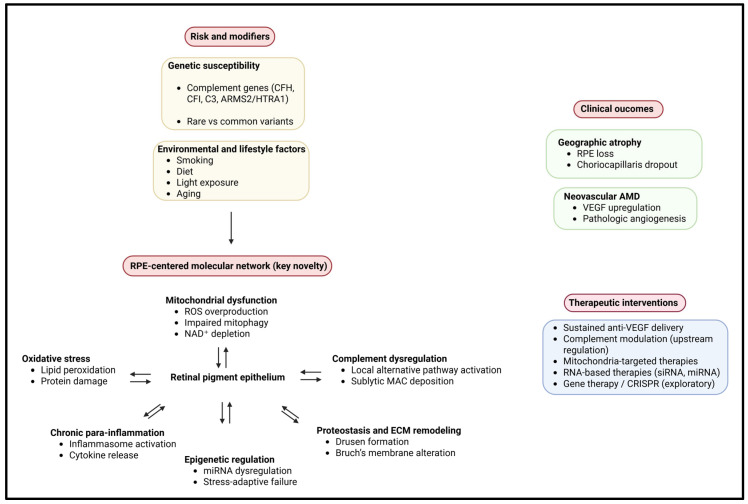
Genetic and environmental risk factors interact at the level of the retinal pigment epithelium to create a self-reinforcing pathogenic network, which is characterized by mitochondrial dysfunction, oxidative stress, complement dysregulation, chronic para-inflammation, epigenetic alterations, and proteostasis imbalance. Mitochondrial dysfunction and oxidative stress act as upstream drivers that promote complement activation and chronic para-inflammation, while sustained complement activation and inflammatory signaling further exacerbate mitochondrial damage, establishing a bidirectional amplification loop. As the network continues to be disrupted over time, it leads to divergent clinical outcomes of geographic atrophy and neovascular age-related macular degeneration.

This review aims to provide a comprehensive and integrative overview of the molecular mechanisms underlying AMD pathogenesis, with a particular focus on the interplay between genetic susceptibility, oxidative stress, mitochondrial dysfunction, inflammation, and epigenetic regulation. Furthermore, we explore how these insights are shaping current and emerging therapeutic strategies, highlighting novel molecular targets and preventive approaches that may ultimately transform AMD management from reactive treatment to proactive disease modification. A schematic overview of the network-based molecular mechanisms underlying AMD pathogenesis and their therapeutic implications is illustrated in Figure 1.

## 2. Genetic Architecture of Age-Related Macular Degeneration

From a genetic standpoint, AMD is a multifaceted and polygenic disorder. Importantly, genetic predisposition alone is insufficient to cause disease. Instead, it interacts dynamically with aging-related processes and environmental exposures to determine disease onset, progression, and phenotype. This complex interplay has direct implications for risk prediction, disease stratification, and the development of targeted therapeutic strategies.

### 2.1. Complement Pathway Polymorphisms as Central Drivers of AMD Risk

Dysregulation of the alternative complement pathway represents the most robust and reproducible genetic signal in AMD. The complement factor H (*CFH*) gene is the principal contributor to genetic risk, with the Y402H polymorphism (rs1061170) conferring a two- to fourfold increased risk of AMD depending on zygosity [7,17]. This amino acid substitution impairs CFH binding to heparan sulfate proteoglycans, C-reactive protein (CRP), and malondialdehyde-modified lipids at the level of Bruch’s membrane and the RPE, resulting in insufficient local complement inhibition and chronic inflammatory activation [18,19].

Additional common variants in complement component genes further modulate disease susceptibility. The C3 R102G variant (rs2230199) enhances complement activation and has been associated with increased drusen burden and progression to advanced disease [7]. Similarly, polymorphisms in complement factor I (CFI), complement factor B (CFB), and complement component 2 (C2) influence complement regulation, either exacerbating or mitigating AMD risk depending on their functional consequences [20]. Protective variants in CFB and C2 reduce alternative pathway amplification, reinforcing the centrality of complement balance in retinal homeostasis.

Collectively, these findings support a model in which AMD arises, in part, from chronic, low-grade complement overactivation at the retina-choroid interface, rather than from episodic inflammatory insults. This genetic framework has directly guided therapeutic development, leading to clinical trials of complement inhibitors targeting C3, C5, and upstream regulatory components for geographic atrophy [21,22]. However, interindividual variability in treatment response highlights the need for genetically informed patient stratification.

### 2.2. The ARMS2/HTRA1 Locus: Multifaceted Effects Beyond Complement

The second most important genetic risk region for AMD is located on chromosome 10q26 and includes the *ARMS2* and *HTRA1* genes. Variants within this region are strongly associated with neovascular AMD and GA in several populations, especially variant rs10490924 [7,23]. Although the precise causal gene remains debated, accumulating evidence suggests that dysregulation of high-temperature requirement A serine peptidase 1 (HTRA1) expression plays a central pathogenic role.

HTRA1 encodes a serine protease that plays an important role in the remodeling of ECM, transforming growth factor-beta (TGF-β) signaling, and angiogenesis. High levels of HTRA1 expression due to risk allele(s) may lead to compromised integrity of Bruch’s membrane, allow for choroidal neovascular invasion, and increase risk of oxidative stress [24,25]. In parallel, age-related maculopathy susceptibility 2 (ARMS2) has been implicated in mitochondrial maintenance and oxidative stress responses, suggesting a potential link between this locus and age-related mitochondrial dysfunction in the RPE [26].

Notably, the ARMS2/HTRA1 risk haplotype is associated with earlier disease onset, faster progression, and more aggressive phenotypes, particularly when combined with complement risk alleles, highlighting epistatic interactions within the AMD genetic landscape [2].

### 2.3. Common Versus Rare Variants: Layered Genetic Contributions

In contrast to common variants, which contribute cumulatively to disease susceptibility and are particularly informative at the population level [7], rare variants, often uncovered through whole-exome or whole-genome sequencing, provide mechanistic insights by directly impairing protein function.

Rare loss-of-function variants in CFH, CFI, and C9 significantly increase the risk of AMD by severely compromising complement regulation [27,28]. Compared with common variants, rare variants exhibit higher penetrance, and their penetrance in individuals may be similar to that of an individual with only one variant. Therefore, individuals harboring rare variants within complement systems may present distinct phenotypic features compared to affected individuals with common variants, and thus would provide the optimal group of individuals for the use of targeted/complement-based therapies (or gene therapy).

The co-existence of common and rare variants indicates that the genetic architecture of AMD is complex and multi-level, consisting of a combination of cumulative polygenic risk combined with discrete molecular weaknesses that act together to define disease expression.

### 2.4. Gene-Environment Interactions: Modifiers of Genetic Susceptibility

Genetic risk for AMD is greatly affected by environmental sources. The most significant environmental factor is smoking. It is considered the most influential modifiable risk factor. Smoking causes oxidative stress, impairment of mitochondrial function, and complement activation [29,30]. It acts synergistically with CFH and ARMS2/HTRA1 genotypes to increase the risk of developing AMD as well as the rapidity of progression [29,30]. Cigarettes are particularly harmful to people who carry high-risk complement types. Thus, there is a gene-environment interaction between these two factors.

Other dietary factors do interact with an individual’s genetic predisposition to AMD. Diets containing high levels of antioxidants, omega-3 fatty acids, lutein, and zeaxanthin have been associated with reduced rates of progression to advanced AMD in people with genetic susceptibility [31,32]. Conversely, diets high in saturated fat may worsen dysregulation of lipids and the inflammatory process, thus increasing complement activation driven by genotypes with a complement genetic risk factor.

Light exposure represents an additional environmental modifier, particularly relevant for individuals harboring genetic variants affecting oxidative stress responses and DNA repair. Blue light–induced phototoxicity disproportionately impacts RPE cells with compromised antioxidant defenses, further linking genetic vulnerability to cumulative environmental stress [33].

### 2.5. Implications for Risk Prediction and Therapeutic Targeting

Advances in AMD genetics have enabled the development of polygenic risk scores that integrate common variants across complement, inflammatory, and lipid pathways. These scores demonstrate an increasing predictive accuracy for disease onset and progression, particularly when combined with environmental and lifestyle factors [16,34]. Such tools may facilitate the early identification of high-risk individuals and inform surveillance and preventive strategies.

From a therapeutic perspective, genetic stratification offers a rational framework for developing precision medicine. Patients with a genetic profile suggesting that their AMD may be driven by complement-driven mechanisms may derive even greater benefits from complement inhibitors. In contrast, patients with a genetic profile consistent with oxidative or mitochondrial deficiencies may respond better to treatment based on various mechanisms. Additionally, the new development of gene therapy and CRISPR-like genome editing technologies may eventually allow physicians to repair the genetic defects associated with a high risk of developing AMD or restore an appropriate balance of complement activity in patients who have already been identified as genetically at high risk of developing AMD due to complement-related genetic profiles.

Combining identification of patients by their genetic composition with observations based on molecular phenotyping and environmental characteristics should ultimately provide the framework for AMD management as we transition from later-stage interventions to personalized prevention strategies and early modification of disease progression.

A summary of the primary genetic and molecular factors associated with AMD development and progression, along with their possible effects on treatment and implications for future therapeutic options, is presented in Table 1.

## 3. Oxidative Stress and Mitochondrial Dysfunction

Building on the concepts introduced above, oxidative stress and mitochondrial dysfunction are now accepted as central drivers of AMD pathogenesis, as supported by converging evidence from human donor studies, experimental models, and integrative reviews [8,9,35]. The RPE is especially vulnerable to mitochondrial damage because it has one of the highest metabolisms, experiences long-term exposure to visible light, has high levels of oxygen, and consistently performs the activity of phagocytosing photoreceptor outer segments that are very high in polyunsaturated fatty acids [8,9,36]. Age-related impairment of mitochondrial homeostasis in RPE cells precedes overt retinal degeneration and provides a unifying mechanistic link between genetic susceptibility, environmental stressors, and downstream inflammatory and complement-mediated damage (as demonstrated in human donor eyes and experimental animal models).

### 3.1. RPE Mitochondrial Damage as an Initiating Event

Proper mitochondrial function is vital for RPE survival. They supply adenosine triphosphate (ATP) via oxidative phosphorylation, maintain Ca^2+^ levels in the cytosol, act as mediators of redox signaling, and participate in lipid metabolism. Defects in both mitochondrial function and structure are present in RPE before the clinical manifestation of AMD (based primarily on analyses of human donor tissue) [8,35]. Transmission electron microscopy was used to assess the mitochondrial ultrastructure in the mitochondria of RPE from AMD donors. Both included several morphological features, with many mitochondria having abnormal amounts of cristae per mitochondrion, a lower degree of mitochondrial DNA, and a greater amount of deletion from mitochondrial DNA than in normal healthy individuals [37,38].

These deleterious changes ultimately lead to increased mitochondrial ATP depletion and increased electron leakage from the electron transport chain. The outcome of these alterations is an overall increase in the generation of metabolic reactive oxygen species. Importantly, damage to the mitochondrial DNA is particularly harmful because of its limited repair capacity and because the mitochondrial DNA is located close to sites of ROS production. The increased accumulation of mitochondrial DNA damage further disrupts the ability of mitochondria to produce ATP, creating a cycle of bioenergetic impairment and oxidative stress [39].

Experimental animal and in vitro models support the concept that mitochondrial dysfunction is sufficient to induce AMD-like phenotypes. Conditional disruption of mitochondrial transcription factor A (TFAM) or components of the electron transport chain in RPE cells leads to progressive RPE degeneration, photoreceptor loss, and inflammatory activation resembling dry AMD and geographic atrophy [40]. These findings strongly suggest that mitochondrial damage is a driver of retinal degeneration, rather than a mere consequence, consistent with recent consensus reviews positioning mitochondrial dysfunction as an early and causative event in AMD pathogenesis. Collectively, these findings support a model in which mitochondrial dysfunction represents an initiating and amplifying event in AMD pathogenesis, preceding overt retinal degeneration and inflammatory escalation [8,9].

### 3.2. Reactive Oxygen Species, Lipid Peroxidation, and Impaired Mitophagy

High levels of ROS are produced by dysfunctional RPE mitochondria, causing negative effects on the rest of the cell. ROS cause lipid peroxidation in both the outer segment membranes of the photoreceptors and in the lipofuscin components of the RPE, making them highly reactive to form aldehydes that are much more toxic, namely malondialdehyde (MDA) and 4-hydroxynonenal (4-HNE) [19]. The products of these reactions subsequently interact with proteins and nucleic acids, leading to cellular dysfunction and innate immunity activation.

The pathological features of AMD involve the accumulation of oxidative damage within the drusen and Bruch’s membrane, linking mitochondrial stress to extracellular pathology [41]. Oxidatively modified lipid and protein molecules are also present in higher concentrations in AMD than in non-AMD patients, and the resulting modified molecules represent damage-associated molecular patterns (DAMPs) for both inflammatory and complement activation pathways.

Under normal circumstances, we have a mechanism for clearing damaged mitochondria from cells, known as mitophagy, which involves the selective removal of damaged mitochondria from the cellular pool through autophagy. This process is regulated by the PTEN-induced kinase 1 (PINK1) and Parkin pathways. With advancing age and chronic oxidative stress, mitophagic flux is impaired in RPE cells, resulting in the accumulation of dysfunctional mitochondria and reinforcing mitochondrial dysfunction as an upstream pathogenic mechanism rather than a secondary effect [8,9,10,42]. Defective mitophagy exacerbates ROS production and cellular senescence, promoting a proinflammatory and prodenerative RPE phenotype.

Recent studies have demonstrated reduced expression and activity of key mitophagy regulators, including PINK1, Parkin, and BCL2-interacting protein 3 (BNIP3), in RPE cells derived from AMD patients [10]. Moreover, impaired lysosomal function, another hallmark of aging RPE, further compromises autophagic and mitophagic clearance, reinforcing mitochondrial dysfunction and oxidative damage [43].

### 3.3. Crosstalk Between Mitochondrial Dysfunction and Complement Activation

The emerging model for AMD pathogenesis involves communication and interaction between the two processes of inflammation caused by high oxidative stress in the eye and damaged/altered mitochondrial function. Mitochondrial injury due to oxidative stress allows mitochondrial DNA (mtDNA), cardiolipin, and oxidized lipids to be released into the cytosol and outside the cell, all of which help initiate and activate innate immune responses [44,45]. When these oxidized mitochondrial components are released, they trigger and accelerate complement activation via the alternative pathway. Complement regulation in the eye is particularly disrupted by oxidative stress-induced mitochondrial injury at the interface between the RPE and Bruch’s membrane.

Conversely, excessive complement activation exacerbates this mitochondrial dysfunction. Sublytic deposition of the membrane attack complex (MAC) on RPE cells induces calcium influx, mitochondrial depolarization, and further ROS generation, even in the absence of overt cell lysis [12].

Notably, genetic variants in complement regulatory genes, such as *CFH* and *CFI*, may exacerbate this process by reducing the capacity to neutralize oxidative stress–induced complement activation, thereby directly linking genetic susceptibility to mitochondrial pathology [19].

### 3.4. Therapeutic Implications: Targeting Mitochondrial Dysfunction

Given the central role of mitochondrial dysfunction in AMD, therapeutic strategies aimed at restoring mitochondrial homeostasis represent a promising avenue for disease modification, particularly in early and intermediate stages.

#### 3.4.1. Mitochondria-Targeted Antioxidants

Most common antioxidant supplement products do not substantially affect the course of AMD. This is likely due to the failure of these supplements to adequately address the ROS generated in the mitochondria. Other antioxidants that specifically penetrate the mitochondria and accumulate before oxidizing mitochondrial ROS include MitoQ, SkQ1, and SS-31 [46,47]. Preclinical trials (primarily in vitro and animal studies) have shown that these compounds lower oxidative stress in RPE cells, maintain mitochondrial morphology, and preserve RPE cell viability under stressful conditions [47]. Although clinical data on AMD remain limited, these compounds represent a rational strategy to address mitochondrial oxidative stress more effectively than systemic antioxidants.

#### 3.4.2. Modulation of NAD^+^ Metabolism

Nicotinamide adenine dinucleotide (NAD^+^) is important for supporting the cellular metabolic processes of the mitochondria, repairing DNA, and mediating sirtuin responses to cellular stress with age. The aging eye has shown decreased levels of NAD^+^ in the retina, and this decline is directly linked to increased oxidative damage and decreased mitochondrial function [48]. Restoring cellular NAD^+^ has been shown to increase both mitochondrial biogenesis and mitophagy. Protecting RPE from oxidative stress can be achieved by providing dietary supplements with specific precursors of NAD^+^ [as in the case of nicotinamide riboside (NR) and nicotinamide mononucleotide (NMN)] or by increasing intracellular NAD^+^ availability [49].

NAD^+^ metabolism intersects with the inflammatory and complement activation pathways in the body, indicating an opportunity for enhanced mitochondrial function through supplementation with NAD^+^, along with benefits to the immune system and other disease states [50].

#### 3.4.3. Mitophagy Enhancers

Mitophagy enhancers are potential therapeutic agents for restoring mitochondrial quality control. Agents that target the PINK1-Parkin pathway stimulate autophagy flux and improve lysosomal dysfunction, demonstrating protective effects against retinal degeneration in animal models [9,10,51]. Notably, growing evidence suggests that impaired autophagy is not limited to selective mitophagy but is instead a wider pathogenic pathway for AMD, leading to the accumulation of damaged proteins, oxidized lipids, and disabled organelles in RPE cells [10,52].

Compounds such as spermidine or urolithin A, which promote mitophagy and autophagy, have gained much interest as possibly being disease-modifying for age-related diseases such as AMD. Through increased autophagic flux, these compounds can help restore proteostasis, restore lysosomal degradation, increase cellular resilience to long-term oxidative damage, and address several pathogenic pathways simultaneously [8,10]. Additionally, improving mitophagy may promote the removal of damaged mitochondria, thus reducing oxidative stress, decreasing inflammatory signals, stopping the cycle of mitochondrial dysfunction, and preventing complement activation [12,19].

Recent experimental data derived from in vivo animal models also support the therapeutic relevance of autophagy modulation in AMD. For instance, Zhang et al. [53] demonstrated that pharmacologically enhancing autophagy regulates several factors that positively affect the integrity of mitochondrial function, production of ROS, and protection of the viability of RPE cells in response to oxidative stress in mouse models (the most commonly used animal model for studying the disease process of dry AMD), suggesting that the regulation of the autophagic process is a potential new therapeutic target to slow the progression of AMD and increase cellular function.

In summary, the data support the speculation that activating or enhancing autophagic/mithophagic activity may also serve as upstream disease-modifying agents that can be used in the early and intermediate stages of AMD when healthy RPE cells exist but are ultimately functionally impaired due to chronic oxidative stress [8,9,10]. Furthermore, because autophagy modulation reduces cellular stress signals that drive chronic para-inflammation and accelerate complement overactivation, it may also complement therapy with either complement inhibition or antioxidant therapy [11,12,19].

## 4. Inflammation and Complement Dysregulation

AMD’s primary pathogenic driver is chronic inflammation, which integrates genetic susceptibility with upstream cellular stress pathways. The difference between AMD and other classical inflammatory diseases is that AMD is associated with chronic low-grade inflammatory processes that develop over many years before culminating in an eventual and dramatic shift to pronounced immune-mediated injury.

### 4.1. Chronic Para-Inflammation Versus Acute Inflammation in AMD

The concept of para-inflammation has been introduced to describe the adaptive, low-level inflammatory response that maintains tissue homeostasis under chronic stress conditions such as aging and metabolic burden [54]. In the aging retina, para-inflammation serves a protective function by facilitating debris clearance, modulating oxidative stress, and maintaining immune surveillance. However, when persistent cellular stress exceeds the buffering capacity of para-inflammatory mechanisms, the response becomes maladaptive [11,12].

In AMD, the ongoing para-inflammatory state is the result of the constant activation of RPE cells, microglia, and choroidal macrophages, all of which produce complement proteins, cytokines, and chemokines continuously, as opposed to acute inflammation states that are temporary, self-limiting, and ultimately result in full recovery of pathogen clearance or tissue repair. In AMD, the absence of a clear initiating pathogen and the persistence of endogenous DAMPs prevent resolution, resulting in a state of chronic inflammatory signaling that promotes tissue degeneration rather than repair [52].

The transition from adaptive para-inflammation to pathological inflammation is now considered a critical inflection point in AMD progression, particularly during the shift from intermediate disease to geographic atrophy or neovascular complications.

### 4.2. Complement Overactivation and Its Role in Drusen Formation

Drusen, the extracellular deposits that hallmark early and intermediate AMD, are now recognized as immunologically active structures rather than inert byproducts of aging. Proteomic analyses of isolated human drusen and donor retinal tissue have revealed that drusen are enriched in complement components and regulators, including C3, C5, factor B, factor H-related proteins, and terminal complement complex constituents [13,41].

Oxidative stress–modified lipids, lipoproteins, and cellular debris accumulating between the RPE and Bruch’s membrane act as potent activators of the alternative complement pathway. In genetically susceptible individuals, particularly those carrying risk variants in CFH, CFI, or C3, local complement regulation is insufficient, leading to sustained complement amplification at the retinal interface [20,27].

Complement activation products, such as C3a and C5a, further recruit and activate immune cells, enhance vascular permeability, and stimulate angiogenic signaling, thereby linking drusen-associated inflammation to both geographic atrophy and neovascular AMD. Importantly, sublytic deposition of the MAC on RPE cells induces cellular stress responses without immediate lysis, thereby contributing to RPE degeneration [55].

### 4.3. Local Versus Systemic Complement Activation: Distinct but Interconnected Processes

A critical question in AMD pathogenesis concerns the relative contributions of local ocular versus systemic complement activation. While elevated systemic complement activation markers have been detected in AMD patients, mounting evidence suggests that local complement dysregulation within the retina and choroid is the dominant driver of disease pathology [56].

The eye possesses a semi-autonomous immune environment, with local synthesis of complement components by RPE cells, microglia, Müller glia, and choroidal endothelial cells. This localized production allows for rapid, site-specific complement activation but also necessitates tight regulatory control. Genetic defects in complement regulators disproportionately affect this local system, where compensatory mechanisms are limited [18].

The ability for systemic complement activation to modify disease instead of directly cause it may affect the degree of severity of the disease, whether it is symmetric on both sides, or result in an increased amount of inflammation outside the eye. This has important implications for future therapeutic strategies, as a systemic inhibitor of complement could theoretically be less effective because it would not completely block the pathogenic effect on complement in the retina and may also pose a greater risk of unwanted side effects throughout the body systemically.

### 4.4. Lessons from Complement Inhibitor Trials: Successes and Limitations

The strong genetic and mechanistic evidence implicating complement dysregulation in AMD has driven the development of complement-targeted therapies, particularly for geographic atrophy. Phase 2 and phase 3 human clinical trials targeting C3 and C5 have demonstrated that complement inhibition can slow the rate of GA lesion expansion, validating complement overactivation as a disease-modifying mechanism [21,22].

Pegcetacoplan, a C3 inhibitor, and avacincaptad pegol, a C5 inhibitor, have both shown statistically significant reductions in GA growth rates in phase 3 trials, although these studies differed in molecular target (upstream C3 versus terminal C5 inhibition), dosing frequency, and enrolled patient populations. Notably, the pegcetacoplan trials included a broader GA population with monthly or every-other-month dosing, whereas avacincaptad pegol trials focused on monthly administration and slightly different inclusion criteria regarding lesion characteristics and baseline disease stage. However, these benefits are modest and are accompanied by important limitations. Significant increases in the conversion to nAMD have been reported following treatment with some therapeutic agents, with rates varying between trials depending on the level of complement inhibition, dosing intensity, and baseline patient risk factors, suggesting that they contribute to the rebalancing of unintended pathways that support angiogenesis, raising concerns about their impact on pro-angiogenic states [22].

In addition, complement inhibition does not restore lost vision and retinal tissue but rather provides a disease-modulating effect. The different responses to therapy observed between patients are likely related to genetic differences, pre-existing inflammatory states of the patient, and the stage of disease at the time of treatment.

These trials highlight several key lessons: (i) complement dysregulation is necessary but not sufficient to fully explain AMD progression; (ii) upstream disease drivers, such as oxidative stress and mitochondrial dysfunction, remain active despite complement blockade; and (iii) future strategies may require combination therapies targeting multiple pathogenic axes or genotype-guided patient selection to maximize benefit.

Together, these differences highlight that complement inhibition trials in AMD are not directly interchangeable and should be interpreted in the context of target selection, study design, and patient population characteristics.

## 5. microRNAs and Epigenetic Regulation

While classical epigenetic mechanisms such as DNA methylation and histone modifications primarily regulate gene expression at the transcriptional level, post-transcriptional regulation by microRNAs represents a complementary layer of epigenetic control that fine-tunes gene expression without altering DNA sequence or chromatin structure. In regulating gene expression through post-transcriptional mechanisms (e.g., via modulating mRNA stability and translation), miRNAs (i.e., small noncoding RNAs) are important to the biological function of the retina as they maintain cellular homeostasis. They coordinate stress response, provide immune regulation, regulate mitochondrial function, and are involved in remodeling of the ECM. Also, dysregulation of miRNA activities as a result of AMD provides additional evidence of an epigenetic mechanism that connects the process of aging, the environment, and genetics together for disease onset and progression rather than simply resulting from retinal damage [14,15,57]. Due to their ability to simultaneously regulate multiple targets throughout established cellular pathways, miRNA regulatory activities are particularly appropriate for the function of AMD as a multifactorial and networked disease process.

Oxidative stress is the primary mechanism of RPE damage, and the various microRNAs (miRs) related to AMD regulate pathways that influence the cellular senescence process, mitochondrial resilience, and overall redox status. For example, there is evidence that miR-34a modulates age-related oxidative stress susceptibility. When DNA damage and oxidative stress induce the expression of miR-34a, it downregulates sirtuin 1 (SIRT1) expression, a critical regulator of mitochondrial biogenesis, antioxidant responses, and mitophagy. Reduced SIRT1 expression is thought to be responsible for promoting impaired mitochondrial function and increasing the production of reactive oxygen species, thereby contributing to the activation of degenerative signaling mechanisms in RPE cells that result in an increase in oxidative injury [58]. Furthermore, the aberrant regulation of other miRNAs, such as miR-21 and miR-146a, may lead to the aberrant regulation of the oxidative stress response and cell senescence, impairing the ability of retinal cells to respond to environmental changes [59,60]. Based on the collective evidence, we present a model in which miRNA shifts that occur as a result of stress and age promote decreased antioxidant capacity and increased susceptibility to cumulative oxidative damage in RPE cells.

In addition to regulating redox signals, miRNAs are intimately associated with inflammatory and innate immune pathways implicated in AMD. Several studies have considered the role of miRNAs in the genesis and progression of chronic aging-associated para-inflammation, including their mediation of nuclear factor kappa-light-chain-enhancer of activated B cells (NF-kB) signaling, cytokine production, and complement pathways. For example, miR-146a is consistently elevated when challenged by pro-inflammatory signals. It serves as an inhibitory factor by targeting critical intermediates [interleukin-1 receptor-associated kinase 1 (IRAK1) and tumor necrosis factor receptor-associated factor 6 (TRAF6)] and other involved proteins. The primary outcome is to limit the magnitude of the inflammatory response at first. However, continued dysregulation of miR-146a due to chronic stress will ultimately lead to maladaptive, overly strong downregulation, which will lead to the lack of adequate downregulation of inflammatory pathways, the loss of cellular protective mechanisms, and ultimately to the activation of pathways leading to the development of AMD [11,14,57]. The consistent identification of dysregulated miRNAs associated with AMD in ocular fluids, peripheral blood, and retinal tissues suggests that these miRNAs operate at the intersection of crosstalk between complement and inflammation, implicating multiple pathways in the pathogenesis of AMD rather than being limited to a few isolated molecular events [15,61].

miRNAs are essential elements of the angiogenic and extracellular remodeling processes, which are central to the development of neovascularization from intermediate AMD to the fully developed neovascular (NV) phenotype. Regulatory activities occur in endothelial cells of the vascular tissue through regulation via miRNAs of both vascular permeability and matrix composition. Numerous miRNAs modulate endothelial activation and permeability by regulating angiogenic factors, integrins, and ECM components. miR-29a is a compelling example of an miRNA with anti-angiogenic effects in experimental ocular NV models. Conversely, miR-29a alters the expression of genes that encode ECM components. Restoration of miR-29a activity suppresses pathological neovascular growth and influences structural remodeling processes relevant to both Bruch’s membrane integrity and choroidal angiogenesis [62]. More broadly, miRNA families implicated in AMD-related angiogenesis, including miR-21, miR-126, miR-155, and miR-23/27 clusters, regulate interconnected networks that govern endothelial behavior, inflammatory signaling, and tissue remodeling. Although individual miRNA findings vary across studies, network-level convergence on angiogenic and ECM pathways provides biological coherence to these observations [15,57].

The fact that miRNAs are relatively stable in biofluids has led to the development of great interest in using them as biomarkers for minimally invasive procedures. Circulating miRNAs are located within serum or plasma, where they are protected against degradation via encapsulation in extracellular vesicles and also through their attachment to various proteins and lipoproteins. Numerous studies have demonstrated significant differences in the levels of circulating miRNAs when comparing samples taken from patients with AMD and healthy controls, as well as between different AMD subtypes, thus establishing the ability of these circulating miRNAs to potentially provide an appropriate means for risk stratification and/or monitoring disease progression [63]. Simultaneously, profiling of miRNAs in ocular fluids, such as aqueous humor, has uncovered disease-associated profiles that may correlate more directly with local retinal and choroidal processes than systemic measurements [61]. Although there is now a significant body of evidence supporting the idea that miRNAs may be useful in clinical tests, there are significant hurdles to overcome before we see miRNAs integrated into practice, including variations in pre-analytical processing, variations in analytical platforms, differences in normalization strategies, and confounding effects of age and concurrent systemic diseases. Recent integrative reviews have noted that when miRNAs become clinically useful, they will most likely be incorporated into multimodal biomarker panels rather than used as standalone diagnostic tests [15,61].

miRNA modulation is a novel and promising approach in terms of therapeutic applications because it allows for the rebalancing of disease-driving pathways rather than simply being directed to a single “downstream” target or “effector.” Therapeutic miRNA mimics can be used to replace normal healthy (protective) miRNAs that are suppressed or downregulated by diseases. Conversely, antagomiRs or anti-miRs may be used to target and specifically kill the pathogenic miRNAs responsible for sustaining oxidative stress, inflammation, or angiogenesis. Advances in oligonucleotide chemistry (e.g., locked nucleic acids and phosphorothioate) have improved oligonucleotide stability and affinity to their targets; therefore, new delivery systems have been developed to promote high ocular specificity and reduce the incidence of off-target side effects [64]. For AMD, miRNA strategies have the potential to enhance current therapeutic options by inhibiting proangiogenic programs, reducing chronic inflammatory signaling, and improving mitochondrial resilience to stress. Thus, the continued rise in RNA-based therapies in ophthalmology provides a strong platform for the potential success and framework for developing new miRNA-based interventions [65]. While there are still many obstacles to the successful development of miRNA-based therapies for AMD, including defining miRNA cell-type specificity in an extremely complex environment (the retina), determining the best treatment and therapeutic dosing regimen for a slowly progressive disease, and finding clinically relevant endpoints that are robust enough to capture the desired modulation at the network level, miRNA-based therapies have great potential as therapeutic agents for disease modification in AMD rather than merely symptomatic control.

## 6. Proteomics and Systems Biology Approaches

Proteomics and systems biology have allowed researchers to know much more about how the tissues function in terms of proteomics or systems biology, especially how they relate to AMD. Proteomics and systems biology provide detailed insights into specific pathways that cannot be identified using only genomic or transcriptomic data. Genome-wide association studies have identified many risk genes for AMD, and transcriptomics have indicated that genes are expressed differently depending on their environment. Proteomic analyses reveal the amount of each protein present, any modifications made to each protein after translation, how each protein is exported out of the cell, and if the activation of signaling pathways related to that protein has occurred [16].

One of the most influential applications of proteomics in AMD has been the molecular characterization of drusen, the extracellular deposits that define early and intermediate disease stages. Early mass spectrometry-based analyses of isolated human drusen demonstrated enrichment of complement components and regulators, acute-phase reactants, apolipoproteins, amyloid-related proteins, and extracellular matrix constituents, fundamentally redefining drusen as biologically active immunometabolic structures rather than passive accumulations of waste material [41]. Subsequent proteomic and immunohistochemical studies confirmed the presence of complement activation products, including components of the terminal complement complex, alongside oxidative stress-modified proteins and lipid peroxidation adducts, directly linking drusen formation to chronic innate immune activation and mitochondrial-derived oxidative injury at the RPE-Bruch’s membrane interface [13,41,66]. More recent high-resolution proteomic and pathology-driven studies have further expanded the drusen proteome to include proteins involved in inflammasome signaling, lipid handling, and cellular senescence, supporting the concept that drusen represent an extracellular fingerprint of sustained RPE stress and impaired clearance mechanisms rather than a single pathogenic pathway [41].

Proteomics has also provided critical insights into disease mechanisms operating within the RPE, a central integrator of metabolic, inflammatory, and extracellular signaling in AMD. Quantitative proteomic studies of human RPE cells exposed to oxidative stress have demonstrated widespread remodeling of intracellular pathways governing redox balance, mitochondrial function, and proteostasis, accompanied by profound changes in the RPE secretome. Under stress conditions relevant to AMD, the RPE exhibits increased secretion of complement components, pro-inflammatory mediators, matrix metalloproteinases, and angiogenic factors, alongside reduced secretion of proteins involved in extracellular matrix stabilization, antioxidant defense, and immune regulation [67,68]. These secretory alterations provide a mechanistic link between intracellular mitochondrial dysfunction and extracellular pathological processes, including Bruch’s membrane degradation, choriocapillaris dysfunction, immune cell recruitment, and angiogenic permissiveness.

Importantly, proteomic analyses have repeatedly demonstrated discordance between transcriptomic and proteomic changes in stressed RPE cells, particularly for secreted proteins and complement regulators, underscoring the limitations of relying on mRNA abundance as a surrogate for functional pathway activity in AMD [34,67]. This disconnect highlights the importance of post-transcriptional regulation, protein stability, trafficking, and secretion dynamics in shaping disease-relevant molecular environments.

In addition to tissue studies, the proteomics of ocular fluid has created a strong translational link between molecular discovery and clinical application. Analysis of the protein composition of both aqueous (fluid) and vitreous (gel-like) humors from patients with AMD has revealed differences in several protein families that may play a role in inflammation, complement-mediated inflammation, oxidative stress, angiogenesis, and lipid metabolism. The pathways identified in these studies correlate well with the pathogenic mechanisms discovered through the study of retinal tissue [69,70,71]. Additionally, high-dimensional proteomic profiling of the aqueous humor associated with geographic atrophy secondary to AMD has identified candidates that can be sourced from retinal and/or RPE tissues using donor eye expression atlases, thus confirming the biological basis for using these fluids to capture biomarkers [72]. Furthermore, ocular fluid proteomics has demonstrated the potential to predict therapeutic responses in AMD. An example would be a study in which proteomic analysis of the aqueous humor from patients who had received anti-VEGF therapy successfully identified protein signature patterns related to the therapeutic response [73].

The integration of proteomics with genomics and transcriptomics through systems biology approaches has further refined the understanding of AMD as a disease driven by interacting molecular networks rather than isolated pathways. Multi-omics analyses consistently reveal that AMD-associated molecular changes cluster into interconnected modules encompassing complement activation, lipid transport, mitochondrial metabolism, proteostasis imbalance, and extracellular matrix remodeling [16,34]. Network-based modeling has enabled the identification of central hub proteins that integrate signals from multiple pathogenic axes and may therefore represent particularly attractive therapeutic targets. Notably, several of these hubs are not directly predicted by genetic association studies alone, illustrating how proteomics-driven systems biology can uncover novel disease mechanisms beyond inherited risk factors.

A recent and particularly promising advance has been the application of proteome-wide Mendelian randomization and colocalization analyses to prioritize proteins with genetically supported causal roles in AMD. These approaches integrate protein quantitative trait loci with AMD GWAS data to distinguish proteins that are likely drivers of disease from those that are secondary consequences. Recent studies have identified multiple candidate proteins associated with overall AMD risk as well as with dry and neovascular subtypes, and have further assessed their druggability using in silico and chemical annotation frameworks [16]. When interpreted alongside tissue and ocular-fluid proteomics, these causal inference approaches provide a powerful strategy for therapeutic target prioritization.

AMD has been recognized as a result of multiple interacting molecular networks due to genetic predisposition, age-related declines in mitochondrial function and proteostasis, and chronic inflammation through proteomics and systems biology. The understanding of AMD’s molecular makeup through the identification of the chemical compounds found in the deposits of drusen, identification of the changes in the way that RPE secretes material into the bloodstream, and integration of multi-omics data into a direct experimental model has allowed for the discovery of potential new molecular therapeutic targets for treatment as well as the establishment of mechanistic pathways that extend beyond current anti-VEGF and complement-based treatments. The evolution and continued maturation of both spatially resolved proteomic technology and single-cell proteomic technologies, as well as the development of artificial intelligence technologies to develop proteome network analysis, will likely play an important role in the application of precision medicine to AMD, allowing for earlier diagnosis, better modification of the disease, and delaying the onset of severe late-stage AMD.

## 7. Molecular Determinants of Progression from Intermediate Age-Related Macular Degeneration

One of the most clinically and biologically compelling aspects of AMD is the striking heterogeneity in disease progression observed among patients with intermediate AMD. While some individuals remain stable for many years with minimal functional decline, others rapidly progress to late-stage complications, including GA or nAMD. This variability cannot be explained by age or fundus phenotype alone and instead reflects complex interactions among genetic predisposition, molecular stress responses, immune regulation, and environmental modifiers. Understanding the molecular determinants that govern progression from intermediate AMD is therefore central to identifying high-risk individuals and defining preventive therapeutic windows.

At the genetic level, cumulative risk conferred by common variants in complement pathway genes, particularly *CFH*, *CFI*, *C3*, and *ARMS2*/*HTRA1*, strongly influences the likelihood and tempo of progression, but does not fully determine disease fate. Polygenic risk score analyses demonstrate that individuals in the highest genetic risk strata are significantly more likely to progress to advanced AMD, yet substantial overlap exists between progressors and non-progressors, indicating that downstream molecular processes modulate genetic susceptibility [74]. Rare, high-impact variants in complement regulators, especially loss-of-function variants in CFI or CFH, further increase progression risk by amplifying local complement activation and inflammatory stress at the RPE-Bruch’s membrane interface [27]. However, genetics alone is insufficient to explain why progression occurs along divergent trajectories toward GA or neovascular disease.

Studies of human donor eyes and experimental models demonstrate that early mitochondrial impairment in RPE cells precedes overt atrophy and is associated with increased reactive oxygen species production, lipid peroxidation, and accumulation of damaged mitochondria due to defective mitophagy [16]. Importantly, the extent of mitochondrial dysfunction correlates with subsequent RPE loss and GA expansion, suggesting that failure of mitochondrial quality control represents a critical molecular tipping point. Patients with intermediate AMD who exhibit biomarkers of heightened oxidative stress and impaired antioxidant responses appear more likely to progress, particularly toward atrophic phenotypes [7].

Chronic para-inflammation and complement dysregulation represent another major axis influencing progression. In intermediate AMD, low-grade complement activation may initially serve adaptive functions, facilitating debris clearance and immune surveillance. However, sustained complement overactivation, especially in genetically susceptible individuals, drives a transition toward maladaptive inflammation characterized by sublytic membrane attack complex deposition on RPE cells, inflammasome activation, and progressive cellular dysfunction [12]. Evidence from human pathology indicates that local complement activation within the choriocapillaris and RPE precedes capillary dropout and RPE degeneration, processes that strongly associate with GA development [66]. In contrast, complement-derived anaphylatoxins such as C3a and C5a promote vascular permeability and pro-angiogenic signaling, thereby biasing progression toward neovascular conversion in a subset of patients [55].

Lipid metabolism and ECM remodeling further modulate progression risk. Intermediate AMD is characterized by the accumulation of lipid-rich deposits within Bruch’s membrane that impair nutrient diffusion and waste removal. Proteomic and lipidomic analyses reveal that dysregulated cholesterol transport, apolipoprotein accumulation, and oxidative modification of lipids create a pro-inflammatory and pro-angiogenic extracellular milieu [75]. Progressive thickening and calcification of Bruch’s membrane reduce RPE resilience and may favor GA development by exacerbating metabolic stress, whereas focal ECM breakdown and matrix metalloproteinase activity may facilitate choroidal neovascular invasion in susceptible regions [18].

Molecular predictors of GA expansion have been increasingly refined through longitudinal imaging–biomarker correlations and tissue-based studies. Faster GA growth rates are associated with markers of heightened complement activation, mitochondrial dysfunction, impaired autophagy, and RPE senescence [76,77]. Recent clinical trial data further support this framework, as complement inhibition slows GA expansion without restoring lost tissue, implying that complement-driven inflammation acts as a disease accelerator rather than the sole initiator of atrophy [21]. These findings suggest that patients with intermediate AMD who exhibit molecular signatures of oxidative stress, mitochondrial failure, and complement dysregulation may represent optimal candidates for early intervention before irreversible RPE loss occurs.

In contrast, molecular predictors of neovascular conversion emphasize angiogenic permissiveness and inflammatory-vascular crosstalk. Upregulation of VEGF signaling, increased local levels of C3a and C5a, and proteomic signatures indicative of endothelial activation and extracellular matrix degradation have all been associated with a higher risk of neovascular conversion [55]. Importantly, complement inhibition trials have highlighted a critical trade-off: while suppressing complement activity may slow atrophy, it can increase the incidence of neovascular conversion in some patients, underscoring the need for careful molecular stratification and combined pathway targeting [21,77].

Collectively, these insights highlight intermediate AMD as a dynamic molecular state rather than a static clinical category. The concept of preventive therapeutic windows is therefore central to disease modification. Interventions targeting oxidative stress, mitochondrial dysfunction, complement dysregulation, or lipid metabolism are likely to be most effective when applied before self-reinforcing degenerative loops become established [78].

Key molecular predictors associated with progression from intermediate AMD, together with their linked clinical outcomes and translational relevance, are summarized in Table 2.

## 8. Emerging Therapeutic Strategies

The therapeutic landscape of AMD is undergoing a profound transformation driven by the recognition that sustained disease control and prevention of progression will require strategies that extend beyond repeated intravitreal inhibition of VEGF. While anti-VEGF therapy has revolutionized the management of neovascular AMD, treatment burden, variable durability, incomplete response, and the lack of effective therapies for GA underscore the need for innovative approaches that target upstream pathogenic mechanisms, enable long-term pathway modulation, and allow molecularly informed patient stratification.

Sustained anti-VEGF delivery represents a critical evolution in established therapeutic paradigms. The purpose of long-term VEGF inhibition is due to the fact that many intravitreal injections lead to a high burden on both patients and the health system, and that they expose the retina to elevated levels of VEGF during periods of injection and lower levels during periods when injections are not administered. Gene therapy using adeno-associated virus (AAV) viral vectors can be used to provide long-term delivery of VEGF inhibitors after a single administration. Early phase clinical trials of intravitreal or subretinal AAV-mediated expression of anti-VEGF molecules have demonstrated durable intraocular protein expression and reduced need for rescue injections in selected patients, although variability in transgene expression and inflammatory responses remain important challenges [79,80]. In parallel, device-based strategies, such as refillable port delivery systems, provide sustained intraocular release of approved anti-VEGF agents, enabling extended dosing intervals while preserving the ability to discontinue therapy in the event of adverse effects [81]. Together, these approaches highlight a broader shift toward pharmacokinetic stability as a determinant of therapeutic success, rather than simply increasing molecular potency. An overview of emerging therapeutic strategies for AMD, including their molecular targets, mechanisms of action, disease stage applicability, and current limitations, is provided in Table 3.

The complement system is another target for therapeutic intervention in dry AMD and GA. Therefore, it is one of the areas of intense focus for both basic and clinical research on dry AMD and GA. There is a long history of research based on genetic and/or pathological evidence linking chronic complement overactivation to the progression of dry AMD and GA; thus, suppression of complement overactivation remains a focus for future treatment of dry AMD and GA. Recent clinical developments for dry AMD and GA have mainly focused on inhibiting either C3 or C5 in the complement pathway. However, emerging strategies aim to modulate the complement pathway in a specific manner, such that physiological immune surveillance remains unaltered while pathogenic amplification is inhibited. Upstream regulators of the alternative complement pathway, including factor B and factor D, will be targeted for inhibition. Additionally, promotion of endogenous regulation of complement through the use of factor H-based strategies may provide a theoretical advantage in reducing local area dysregulation of complement without complete blockade of the alternative pathway [82]. Early clinical experience suggests that complement inhibition can slow GA lesion expansion, but modest efficacy and increased risk of neovascular conversion in some treatment arms emphasize the need for refined patient selection and potentially combination strategies [22].

Gene therapy strategies for AMD are also expanding beyond anti-VEGF delivery toward correction or compensation of genetic risk alleles. The identification of rare, high-impact variants in complement regulatory genes has provided a compelling rationale for gene augmentation approaches aimed at restoring local complement control. Preclinical studies demonstrate that increasing intraocular expression of complement regulators can reduce complement activation and protect retinal tissues from degeneration, supporting the feasibility of genotype-informed gene therapy [27]. However, the polygenic nature of AMD poses significant challenges, as common risk alleles exert modest effects and interact with environmental and aging-related stressors. Consequently, gene therapy for AMD is likely to be most effective in carefully selected subsets of patients with defined genetic vulnerabilities rather than as a universal intervention.

Genome-editing technologies are currently being developed as potential means of permanently correcting pathogenic genetic variants associated with retinal diseases using CRISPR/Cas systems. The evolving interest in retinal diseases is fueled by the fact that the eye is uniquely suited for gene-editing applications because of the immune-privileged state of the ocular environment, relative compartmentalization, and direct accessibility of the retina. Several proof-of-principle studies support the ability of CRISPR/Cas-based editing to be successfully delivered to retinal cells in living organisms. This raises the possibility of directly targeting genetics associated with AMD or modulating the pathways associated with the development of AMD at the genomic level [83,84]. However, there are still many limitations to the clinical translation of these technologies. These include issues related to the potential for off-target effects, mosaicism, and delivering the most efficient methods of administering CRISPR/Cas directly to post-mitotic retinal cells, and difficulties associated with identifying appropriate genetic targets in complex multifactorial diseases such as AMD, where the majority of risk is due to common variants rather than single genetic causative mutations. These limitations suggest that the application of CRISPR/Cas approaches to AMD is currently at an experimental stage and is more likely to complement, rather than supplant, the use of other therapeutic strategies in the near future.

Another rapidly developing class of treatments widely applicable to AMD is RNA-based therapeutics. Small interfering RNAs (siRNAs) have previously shown clinical viability in ocular indications and allow selective silencing of genes linked to disease. In addition to suppressing VEGF, siRNA techniques are being investigated to target angiogenic signaling pathways, inflammatory mediators, and complement components, providing modular and possibly combinatorial treatment options [85]. Simultaneously, microRNA manipulation is a new approach to rebalance entire pathogenic networks instead of individual genes. The therapeutic delivery of microRNA mimics or inhibitors holds promise for multi-pathway disease modification because dysregulated microRNAs affect oxidative stress responses, inflammatory signaling, mitochondrial function, and angiogenesis [64]. However, significant issues remain regarding tissue selectivity, transport, durability, and off-target effects, necessitating further technical advancement.

## 9. Future Perspectives: Toward Precision Medicine in Age-Related Macular Degeneration

The growing body of molecular, genetic, and systems-level evidence in AMD has made it increasingly clear that the disease cannot be adequately described, or treated, as a single clinical entity. Instead, AMD represents a spectrum of related but biologically distinct conditions that share overlapping phenotypes while differing substantially in underlying molecular drivers, progression trajectories, and therapeutic responsiveness. The transition toward precision medicine in AMD, therefore, hinges on the ability to define biologically meaningful disease endotypes, integrate multi-dimensional data across scales, and translate this knowledge into personalized prevention and treatment strategies.

AMD molecular endotyping is a fundamental step in precision medicine. The variation observed at the molecular level is not adequately captured by conventional categories based on fundus morphology and late-stage outcome. AMD-associated molecular alterations cluster into separate but overlapping networks involving complement dysregulation, mitochondrial dysfunction, oxidative stress, lipid metabolism, ECM remodeling, angiogenic signaling, and cellular senescence, according to multi-omics studies combining genomics, transcriptomics, proteomics, metabolomics, and epigenetic profiling techniques. Importantly, individuals with similar fundus results may have distinctly different levels of pathway activation, which contributes to the differences in treatment responses and progression rates. This is because these molecular fingerprints do not exactly correspond to clinical presentations. According to recent systems biology frameworks, AMD can be categorized into endotypes based on dominant molecular drivers, such as complement-driven inflammatory, mitochondria–oxidative stress, and angiogenic-ECM remodeling endotypes, each of which has unique therapeutic implications [16].

Advances in molecular endotyping are closely linked to the development of personalized prevention strategies. Intermediate AMD represents a critical window during which pathogenic processes are active, but tissue loss remains limited and potentially modifiable. Genetic risk profiling, particularly through polygenic risk scores incorporating common and rare variants, has demonstrated increasing predictive power for disease progression when combined with environmental and lifestyle factors such as smoking, diet, and light exposure [7]. However, genetics alone is insufficient to guide intervention timing or modality. Integrating molecular biomarkers, derived from ocular fluids, blood-based assays, and advanced imaging correlates, with genetic risk holds promise for identifying individuals poised at a biological tipping point before irreversible degeneration occurs [78]. Such stratification could enable targeted lifestyle interventions, antioxidant or mitochondrial-supportive therapies, and early pathway-specific pharmacologic modulation in patients most likely to benefit, while avoiding unnecessary treatment in low-risk individuals.

Precision medicine in AMD will also require a shift from monotherapies to rational combination strategies that address the multifactorial nature of disease progression. The limited effectiveness of single-pathway treatments, such as VEGF suppression alone in neovascular AMD or complement inhibition alone in geographic atrophy, highlights the existence of parallel and compensatory pathogenic pathways [77]. According to systems-level assessments, combining treatments that target complementary pathways, such as immunological regulation, mitochondrial support, and oxidative stress mitigation, may result in additive or synergistic effects, especially when used early in the course of the illness [16]. For instance, neovascular conversion might be decreased while maintaining tissue integrity by combining long-term antiangiogenic tactics with the modification of inflammatory or ECM pathways. Similarly, pairing complement modulation with interventions that enhance mitochondrial quality control may attenuate both inflammatory amplification and metabolic vulnerability in RPE cells.

However, significant translational gaps and unmet needs remain. Validating and standardizing molecular biomarkers for routine clinical use is a significant challenge. Cross-study comparability and regulatory acceptability are hampered by variations in sample collection, analytical platforms, and cohort composition [15,78]. Furthermore, most molecular discoveries have come from cross-sectional studies or late-stage tissues, but longitudinal, stage-specific data are crucial for determining the primary causes of progression rather than its downstream effects. Another critical gap is the integration of molecular data into clinical trial designs. Many past and ongoing trials enroll heterogeneous patient populations based primarily on anatomical criteria, potentially diluting therapeutic signals that might be robust within molecularly defined subgroups [16].

A conceptual framework linking dominant molecular endotypes of AMD with likely progression patterns and optimal therapeutic windows is outlined in Table 4.

Ethical and practical considerations will also shape the future of precision medicine for AMD. Concerns about cost-effectiveness, testing accessibility, and fair implementation in aging populations are raised by genetic and molecular stratification. The long natural history of AMD makes it more challenging to select clinical endpoints sensitive enough to identify early disease modification, rather than late-stage structural alterations. To solve these problems, coordinated efforts spanning basic research, clinical research, regulatory science, and health policy are required.

Although our understanding of the pathophysiology of AMD has advanced significantly, several significant obstacles limit the interpretation and application of current research findings. The extensive use of animal models, especially mouse and rat systems, which lack a true macula and are essentially different from the human retina in terms of photoreceptor distribution, immunological environment, and complement regulation, represents a significant obstacle. These models have been essential for mechanistic research; however, they are still unable to accurately capture important aspects of human AMD, particularly chronic para-inflammation and geographic atrophy. As a result, preclinical results must be carefully extrapolated to human diseases [86].

The second drawback is the variability in pre-analytical processing and multi-omics approaches. Reproducibility and cross-study comparability can be significantly affected by variations in tissue procurement, postmortem delay, sample preservation, sequencing and proteomic platforms, normalization techniques, and bioinformatic pipelines. This methodological heterogeneity makes the identification of strong molecular signatures more difficult and has been identified as a significant obstacle to the reproducibility and clinical application of multi-omics findings [87,88].

Finally, despite the appealing prospects of microRNA-based approaches for network-level disease modulation, there are substantial obstacles to their clinical implementation. Achieving effective and cell-type-specific delivery within the retina, guaranteeing the longevity of therapeutic effects in a slowly progressing disease, reducing off-target interactions, and resolving long-term safety issues are some major obstacles. In the larger field of RNA-based therapies, these translational barriers are well recognized, and they are especially pertinent to long-term retinal conditions, such as AMD [89].

## 10. Conclusions

Decades of studies have shown that AMD is a complex, network-based disease in which environmental stressors, aging-related cellular decline, and genetic vulnerability all interact with linked molecular systems. Instead of functioning independently, oxidative stress, mitochondrial dysfunction, chronic para-inflammation, complement dysregulation, lipid metabolism imbalance, extracellular matrix remodeling, and aberrant angiogenic signaling form self-reinforcing circuits that change and vary among individuals. This network perspective offers a paradigm for balancing the variety of clinical manifestations, development paths, and treatment outcomes observed in AMD.

Viewing AMD as a network disease has profound implications for therapeutic development. The limited and often variable efficacy of single-pathway interventions, whether anti-VEGF therapy in nAMD or complement inhibition in geographic atrophy, reflects the capacity of interconnected molecular networks to compensate when one node is suppressed. Sustained disease control and true modification of disease course are therefore unlikely to be achieved through isolated targeting of downstream effectors alone. Instead, integrated molecular targeting strategies that address upstream drivers and multiple converging pathways will be required. Advances in genomics, epigenetics, proteomics, and systems biology have begun to identify such convergence points, including mitochondrial quality control, complement regulation, and RPE stress signaling, which may represent leverage nodes capable of rebalancing pathogenic networks rather than merely dampening their outputs.

Equally important is the recognition that therapeutic timing is as critical as the therapeutic target. The typical focus on late-stage AMD, when significant retinal pigment epithelium and photoreceptor loss occurs, reduces the possibility of functional recovery. In contrast, intermediate AMD is a physiologically active but theoretically modifiable stage in which the tissue architecture is mostly unaltered, yet pathogenic processes continue. According to molecular profiling and endotyping, there are critical therapeutic windows during which interventions can delay, stop, or reverse the progression of the illness. To change the therapeutic paradigm from late-stage rescue to early disease modification, validated molecular biomarkers, better risk classification, and trial designs that link treatment approaches to the underlying illness biology rather than morphological aims are necessary.

To summarize, the primary findings of the present review are as follows: (i) AMD is a networked disease characterized by mitochondrial dysfunction, oxidative stress, complement dysregulation and inflammation, epigenetic control mechanisms, and dysregulation of proteostasis; (ii) an early component of AMD pathogenesis is the failure of mitochondrial function upstream and reduced stress resilience in RPE cells. This leads to a resultant increase in inflammatory and complement-mediated injury. (iii) Inter-individual molecular heterogeneity is an important factor in determining the differences in prognosis and response to therapy associated with the development of AMD. Finally, advances in managing AMD in clinical practice will depend on the incorporation of multi-omics-based molecular endotyping, validated biomarkers, and genetic risk assessment to enhance decision-making in designing future clinical trials for AMD. These advances will facilitate the use of precision medicine to provide evidence-based early intervention, the development of rational combination therapies that target multiple pathogenic pathways, and the transition from late-stage rescue to proactive disease modification and prevention.

## Figures and Tables

**Table 1:** Genetic and Molecular Determinants of AMD Pathogenesis and Progression.

Molecular Factor	Key Genes/Pathways	Functional Impact	Association with Disease Stage	Primary Evidence Type	Therapeutic Implications
Complement regulation	CFH, CFI, C3, CFB, C2	Impaired control of the alternative complement pathway; increased local inflammation	Early and intermediate AMD; GA progression	Human genetics (GWAS); human pathology; clinical trials	Complement inhibitors; gene augmentation strategies
Oxidative stress response	SIRT1, NRF2 pathway	Reduced antioxidant defense and stress resilience	Intermediate AMD; GA expansion	Experimental models; human donor tissue	Mitochondria-targeted antioxidants; NAD^+^ modulation
Mitochondrial quality control	PINK1, PARKIN, mitophagy pathways	Accumulation of dysfunctional mitochondria; increased ROS	Early RPE dysfunction; GA	In vitro studies; animal models; donor eye analyses	Mitophagy enhancers; mitochondrial stabilizers
Angiogenic signaling	VEGF, HIF-1α	Pathological neovascularization	Neovascular AMD	Human clinical trials; experimental models	Anti-VEGF, sustained delivery systems
Lipid metabolism	APOE, HDL-related pathways	Lipid accumulation in Bruch’s membrane	Early/intermediate AMD	Human genetics; proteomics; observational studies	Lipid-modulating therapies

Abbreviations: AMD, age-related macular degeneration; CFH, complement factor H; CFI, complement factor I; C3, complement component 3; CFB, complement factor B; C2, complement component 2; GWAS, genome-wide association study; SIRT1, sirtuin 1; NRF2, nuclear factor erythroid 2–related factor 2; ROS, reactive oxygen species; PINK1, PTEN-induced kinase 1; PARKIN (PRKN), parkin RBR E3 ubiquitin protein ligase; VEGF, vascular endothelial growth factor; HIF-1α, hypoxia-inducible factor 1 alpha; APOE, apolipoprotein E; HDL, high-density lipoprotein; RPE, retinal pigment epithelium; GA, geographic atrophy.

**Table 2:** Molecular Predictors of Progression from Intermediate AMD.

Predictor Category	Molecular Features	Associated Outcome	Evidence Type	Clinical Relevance
Genetic risk burden	High polygenic risk score; rare complement variants	Faster progression to advanced AMD	GWAS; longitudinal cohorts	Risk stratification; preventive targeting
Complement activation	Elevated local MAC deposition; C3a/C5a signaling	GA expansion or neovascular conversion	Human pathology; clinical trials	Complement modulation
Mitochondrial dysfunction	Reduced mitochondrial mass; impaired mitophagy	RPE degeneration; GA	Donor eyes; preclinical models	Mitochondria-targeted therapies
Inflammatory signaling	Chronic para-inflammation; inflammasome activation	Accelerated progression	Tissue studies; biomarkers	Anti-inflammatory strategies
ECM remodeling	MMP activation; Bruch’s membrane thinning	Neovascular conversion	Proteomics; imaging correlations	Anti-angiogenic combination therapies

Abbreviations: AMD, age-related macular degeneration; GA, geographic atrophy; MAC, membrane attack complex; C3a, complement component 3a; C5a, complement component 5a; RPE, retinal pigment epithelium; ECM, extracellular matrix; GWAS, genome-wide association studies.

**Table 3:** Emerging Therapeutic Strategies in AMD beyond Conventional Anti-VEGF.

Therapeutic Strategy	Molecular Target	Mechanism of Action	Disease Stage	Key Limitations
Sustained anti-VEGF delivery	VEGF	Continuous intraocular VEGF suppression	Neovascular AMD	Inflammation; device or vector-related risks
Complement modulation (upstream)	Factor B, Factor D, CFH	Attenuation of alternative pathway amplification	GA, intermediate AMD	Neovascular risk; modest efficacy
Gene therapy	Complement regulators, VEGF	Long-term expression of therapeutic proteins	Selected AMD subtypes	Irreversibility; patient selection
CRISPR/Cas editing	AMD-associated risk loci	Permanent genomic modification	Experimental	Off-target effects; delivery challenges
RNA-based therapies	VEGF, complement genes, miRNAs	Post-transcriptional gene regulation	Neovascular and dry AMD	Durability; tissue specificity

Abbreviations: AMD, age-related macular degeneration; VEGF, vascular endothelial growth factor; GA, geographic atrophy; CRISPR, clustered regularly interspaced short palindromic repeats; Cas, CRISPR-associated endonuclease; RNA, ribonucleic acid; RNA; miRNA, microRNA.

**Table 4:** Toward Precision Medicine in AMD: Molecular Endotypes and Therapeutic Windows.

AMD Molecular Endotype	Dominant Pathways	Likely Progression	Optimal Intervention Window	Therapeutic Approach
Complement-driven inflammatory	Alternative complement activation	GA > neovascular	Early–intermediate AMD	Complement modulation
Mitochondrial–oxidative stress	ROS accumulation; mitophagy failure	GA	Intermediate AMD	Mitochondrial support
Angiogenic–ECM remodeling	VEGF signaling; ECM breakdown	Neovascular AMD	Late intermediate AMD	Anti-VEGF ± ECM targeting
Mixed endotype	Multiple overlapping pathways	Variable	Individualized	Combination therapy

Abbreviations: AMD, age-related macular degeneration; GA, geographic atrophy; ECM, extracellular matrix; ROS, reactive oxygen species; VEGF, vascular endothelial growth factor.

## Data Availability

No new data were created or analyzed in this study.

## Abbreviations

The following abbreviations are used in this manuscript:

AMD	Age-related macular degeneration
AAV	Adeno-associated virus
APOE	Apolipoprotein E
ARMS2	Age-related maculopathy susceptibility 2
ATP	Adenosine triphosphate
BNIP3	BCL2-interacting protein 3
Cas	CRISPR-associated endonuclease
C2	Complement component 2
C3	Complement component 3
C3a	Complement component 3a
C5	Complement component 5
C5a	Complement component 5a
CFB	Complement factor B
CFH	Complement factor H
CFI	Complement factor I
CRISPR	Clustered regularly interspaced short palindromic repeats
CRP	C-reactive protein
DAMPs	Damage-associated molecular patterns
ECM	Extracellular matrix
GA	Geographic atrophy
GWAS	Genome-wide association studies
HDL	High-density lipoprotein
HIF-1α	Hypoxia-inducible factor 1 alpha
HTRA1	High-temperature requirement A serine peptidase 1
IRAK1	Interleukin-1 receptor-associated kinase 1
MAC	Membrane attack complex
MDA	Malondialdehyde
miRNA	MicroRNA
MMPs	Matrix metalloproteinases
mtDNA	Mitochondrial DNA
NAD^+^	Nicotinamide adenine dinucleotide
NF-κB	Nuclear factor kappa-light-chain-enhancer of activated B cells
NMN	Nicotinamide mononucleotide
NR	Nicotinamide riboside
nAMD	Neovascular age-related macular degeneration
NV	Neovascular
PINK1	PTEN-induced kinase 1
PARKIN (PRKN)	Parkin RBR E3 ubiquitin protein ligase
RPE	Retinal pigment epithelium
RNA	Ribonucleic acid
ROS	Reactive oxygen species
siRNA	Small-interfering RNA
SIRT1	Sirtuin 1
TGF-β	Transforming growth factor beta
VEGF	Vascular endothelial growth factor
